# Breast Implant-associated Anaplastic Large Cell Lymphoma Affecting a Neosubpectoral Pocket

**DOI:** 10.7759/cureus.7178

**Published:** 2020-03-04

**Authors:** Greg Hobson, John Bates, Daniel Sherbert

**Affiliations:** 1 Plastic Surgery, Beaumont Health, Farmington Hills Campus, Royal Oak, USA; 2 Plastic Surgery, Beaumont Health, Royal Oak, USA

**Keywords:** alcl, bia alcl, anaplastic large cell lymphoma, breast implant associated anaplastic large cell lymphoma, neosubpectoral

## Abstract

The complications associated with breast implants are under perpetual scrutiny to maximize patient safety. In this era of plastic surgery, a new concern being addressed is breast implant-associated anaplastic large cell lymphoma (BIA-ALCL). Patients with BIA-ALCL most commonly present several years after implant placement with a periprosthetic fluid collection. The body of literature and reports of BIA-ALCL continues to grow with additional nuances in proposed causes as well as management. Most recently, this has led to a recall of breast implants manufactured utilizing a specific texturing. We describe here the time course, diagnosis, and management of BIA-ALCL in a 52-year-old patient who underwent submuscular implant-based reconstruction and subsequent revision of that reconstruction with the creation of a neosubpectoral pocket. The patient was managed in accordance with current guidelines under the supervision of a multidisciplinary team. In our review of the literature, several case reports, case series, and guideline publications were identified. Current guidelines for management are based on the staging of BIA-ALCL at diagnosis and span from only surgical with implant removal, excision of the lymphoma, and excision of the surrounding fibrous capsule to addition of chemotherapeutic regimens or radiation for distant and locally advanced disease.

## Introduction

Breast implant-associated anaplastic large cell lymphoma (BIA-ALCL) is a rare T-cell lymphoma affecting patients with textured breast implants. It is characterized by a delayed fluid collection surrounding an implant that when evaluated by cytology demonstrates high expression of the CD30 cell surface protein [1].

The National Comprehensive Cancer Network (NCCN) has published guidelines for the diagnosis, management, and treatment of BIA-ALCL and classified BIA-ALCL stages according to classic TNM style staging [1]. The management primarily consists of surgical excision of the lymphoma with removal of the associated implant and capsule with higher levels of disease being considered for chemotherapy or radiation. This represents an obvious concern for all patients with implants placed for cosmetic or reconstructive purposes. The United States Food and Drug Administration has recently requested that Allergan recall all implants that undergo a specific type of texturing during its manufacturing process [2].

As the body of literature and information continues to grow on BIA-ALCL, we describe a patient who was diagnosed and treated for BIA-ALCL affecting a neosubpectoral pocket.

## Case presentation

A 52-year-old female underwent bilateral simple mastectomy with left sentinel node biopsy in October 2014 for left sided invasive ductal carcinoma. Pathology demonstrated a 0.8-cm focus of invasive disease with an adjacent 1.1-cm focus of ductal carcinoma in situ of the left breast and no disease detected in the three sentinel lymph nodes. She had stage 1 disease, T1bN0M0, according to NCCN TNM staging. As no radiation was indicated for her breast cancer, she underwent placement of bilateral textured subpectoral tissue expanders (Mentor Contour Profile Siltex, Mentor Worldwide LLC, Irvine, CA) with acellular dermal matrix (SurgiMend, Integra Life Sciences, Plainsboro Township, NJ ). A minimal touch technique was used, and the subpectoral pocket was irrigated with antibiotic solution consisting of bacitracin and polymyxin. 

Following an uneventful expansion process in April 2015, she was taken to the operating room for implant exchange. Due to oversized subpectoral pockets, a neosubpectoral plane was created between the anterior capsule and pectoralis major. The former pocket was obliterated with a combination of cautery and monofilament absorbable suture. After irrigating with triple antibiotic solution, Mentor Contour Profile textured silicone implants (Mentor Contour Profile) were then inserted bilaterally using a no-touch technique.

**Figure 1:**
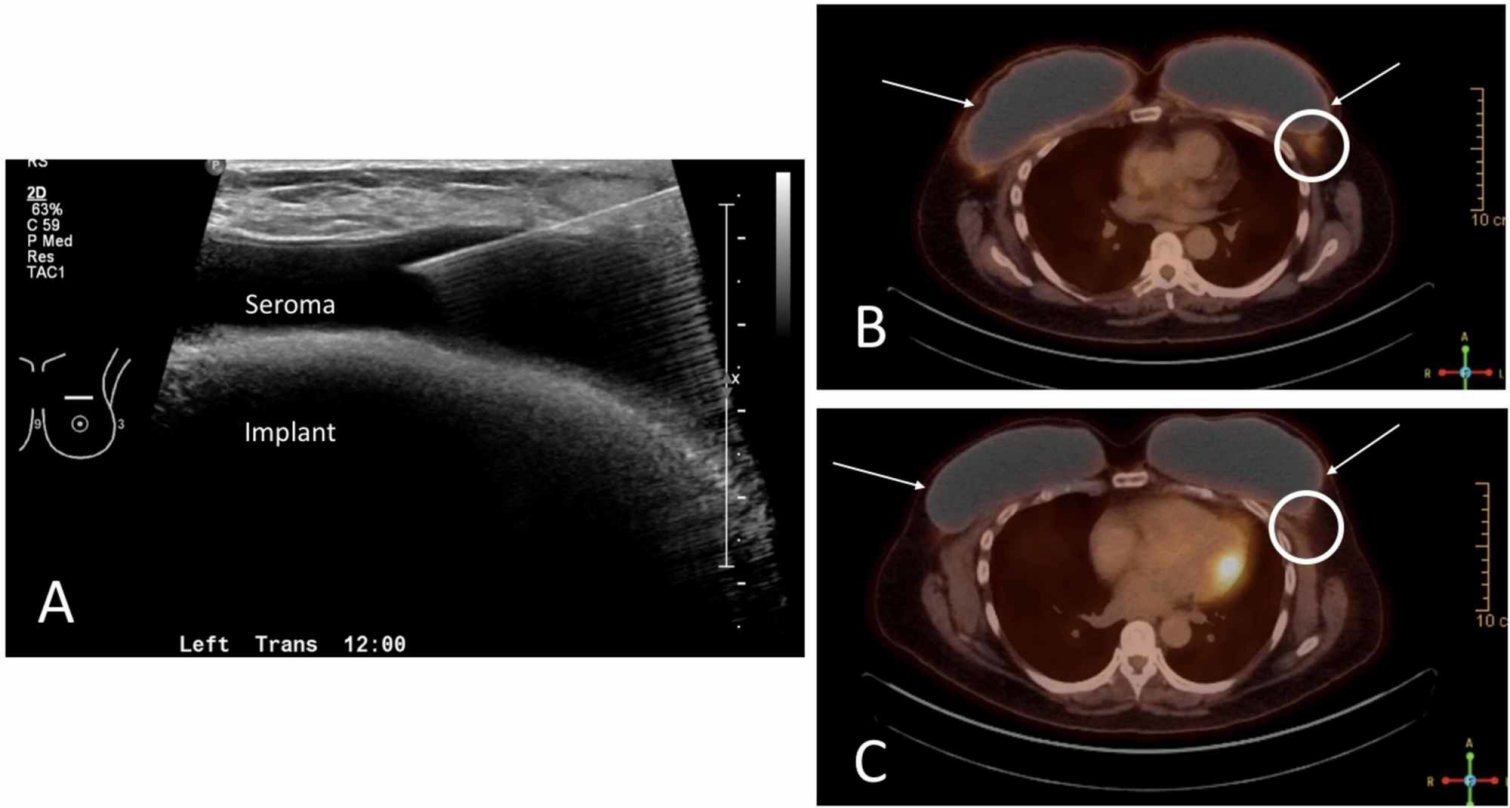
Perioperative imaging (A) Ultrasound-guided aspiration periprosthetic seroma for diagnosis of BIA-ALCL. (B) Immediate postoperative PET scan demonstrating postoperative increased uptake at the surgical site (white arrows) and reactive lymph nodes in the left axilla (white circle). (C) One-year interval PET scan demonstrating reduced surgical site uptake (white arrows) and improved reactive lymph nodes in the left axilla (white circle). BIA-ALCL, breast implant-associated anaplastic large cell lymphoma; PET, positron emission tomography.

In June 2018, the patient presented with unilateral left breast swelling and medial rash. An ultrasound was performed identifying a seroma that was aspirated by interventional radiology (Figure 1A). Approximately 250 mL of clear yellow fluid was obtained and sent for cytological analysis including CD30 to evaluate for BIA-ALCL. CD30 was found to be strongly positive, while ALK was negative. A multidisciplinary approach was taken, and it was determined that the patient would undergo staging CT of the thorax, abdomen, and pelvis which was unremarkable other than the known left breast seroma and operative management of her disease. The patient returned to the operating room for bilateral implant removal, total capsulectomies, and placement of smooth implants. Multiple specimens were sent including bilateral seroma fluid, bilateral capsular debris, bilateral complete capsules, and an enlarged lymph node on the left. Pathologic analysis revealed BIA-ALCL in the left breast seroma, BIA-ALCL of the capsular debris on the left, and a soft tissue mass of the granular surface of the left breast capsule consistent with ALCL. Immunohistochemical stains were performed and consistent with ALCL. There was found to be perilymphoid tissue that was also positive for BIA-ALCL. A postoperative positron emission tomography (PET) scan was performed revealing small nodes of the left axilla with mild uptake favoring reactive lymph nodes (Figure 1B). Multidisciplinary evaluation recommended that she undergo radiation for local control of her T4N0M0 disease, which was uneventfully completed. Interval PET scans were negative for recurrence of disease (Figure 1C). Due to ongoing issues with rotation of the implants, the patient elected to convert her reconstruction to autologous tissue. 

## Discussion

The overall risk of BIA-ALCL ranges from 1:1000 to 1:30,000 based on lifetime risk studies from the USA, Canada, the Netherlands, and Australia [3]. There have been 573 global cases of BIA-ALCL with 33 patient deaths [2]. This risk has prompted surgeons and institutions to modify their consent process for breast implant placement. Penn State Hershey Medical Center has published their experience of conveying information about the risk of BIA-ALCL to patients who had previously had a breast implant placed. In that population, 1,284 letters were sent to patients to provide them with information regarding BIA-ALCL as well as a call line for further questions. In the first 47 days, 76 patients (6.4%) called the hotline and nine of sixteen patients with textured implants opted to undergo further surgery after evaluation [4].

Although the exact etiology of the development of BIA-ALCL remains unclear, the current literature has done well to lay out the presentation, diagnosis, and management. Patients with late-onset seromas should undergo aspiration of their seromas and the fluid evaluated for CD30. If positive, the patient should undergo a multidisciplinary staging process and ultimately have their BIA-ACL excised along with the capsule and implant removal with radiation and chemotherapy as adjuncts. 

Unique to this patient, and not discussed in the literature to our knowledge, is the involvement of a neosubpectoral pocket with BIA-ALCL. In 2009, Maxwell described the efficacy of utilizing a neosubpectoral pocket in revisionary breast reconstruction [5]. In this procedure, the preoperative implant is removed and the anterior leaf of the capsule is dissected free of the pectoralis major and secured to the posterior leaf of the capsule functionally creating a site change. This is relevant to BIA-ALCL discussion as it demonstrates the affliction of novel location of disease. 

Literature review

Breast augmentation has been steadily increasing worldwide since the first augmentation procedure in 1962 [6]. In 2015, approximately 1.4 million breast augmentation cases were performed [7,8]. Recognizing this, it is important to review the presentation, diagnosis, and management of BIA-ALCL.

Since the first reported cases, ALCL, a peripheral T-cell lymphoma, represents approximately 2%-3% of all non-Hodgkin’s lymphomas [9]. BIA-ALCL now represents between 9% and 13% of delayed serums [10]. Recent Food and Drug Administration recommendations for textured implant recall cite 573 unique cases with 33 associated deaths attributable to BIA-ALCL [2]. Though BIA-ALCL is considered rare, the cosmetic use of textured breast implants would imply an increase in life-time cancer risk in elective patient procedures. The increased risk in these patients is not insignificant. 

Initial presentation is most commonly due to unprovoked/non-traumatic swelling and pain associated with spontaneous periprosthetic fluid collections [11]. These generally arise 7-10 years following breast augmentation or reconstruction with textured breast implants. To date, there have been no confirmed cases of BIA-ALCL associated with only smooth device implants [6,12]. Other incidental findings have included mass and lymphadenopathy reported in 8%-24% and 4%-12%, respectively [1,6]. In the setting of these finding and exclusion of delayed seroma, trauma, and infection, there should be suspicion for BIA-ALCL. 

Following a newly released NCCN guideline paper by Clemens et al., initial management should include ultrasound examination for fluid collections, breast masses, and lymphadenopathy. Magnetic resonance imaging recommended for equivocal findings [1]. Fine needle aspiration of periprosthetic fluid collection should be collected with a minimum of 50cc sent for cytologic evaluation. Suspicious masses require tissue biopsy, with the understanding that capsular lining may only contain sparse malignant cells and thus may yield a false-negative report [6]. 

Fundamental immunochemistry for BIA-ALCL includes fine needle aspiration with CD30 expression and absence of anaplastic lymphoma kinase [1]. Though this is not diagnostic as several other biomarkers may be present, it should prompt consultation to a tertiary center for definitive diagnosis of BIA-ALCL. 

Following definitive diagnosis, a complete surgical excision is the only definitive therapy [13]. Established surgical guidelines include complete capsular excision including possible associated masses. Orientation for pathological evaluation is encouraged for anatomical description of possible residual disease. Radical mastectomy, sentinel lymph node biopsy, and axillary dissection have not been of proven benefit [1].

Postoperative management is primarily dependent upon surgical margins. If a clear margin is obtained, prognosis is excellent and no indication for adjuvant therapy is warranted. In a study of 87 BIA-ALCL patients, Clemens and Horwitz reported an overall survival rate of 94% and 91% at three and five years, respectively [13]. If extracapsular spread, positive margins, or lymph node involvement is seen, several investigational therapies are currently ongoing. The therapies include radiation and chemotherapy based primarily upon first-line therapies for systemic T-cell lymphoma. 

## Conclusions

BIA-ALCL continues to warrant further study in an effort to elucidate better understanding of the pathogenesis. Recall of the most commonly involved texturing on a breast implant was paramount to promoting patient safety until this disease process is better understood. It is essential to have an open flow of information and document these cases as they occur to determine how BIA-ALCL can be prevented. At 20 months following the diagnosis of BIA-ALCL, the patient remains disease-free and has since underwent autologous breast reconstruction.
